# Molecular characterisation of *Acinetobacter baumannii* isolates from bloodstream infections in a tertiary-level hospital in South Africa

**DOI:** 10.3389/fmicb.2022.863129

**Published:** 2022-08-05

**Authors:** Michelle Lowe, Ashika Singh-Moodley, Husna Ismail, Teena Thomas, Vindana Chibabhai, Trusha Nana, Warren Lowman, Arshad Ismail, Wai Yin Chan, Olga Perovic

**Affiliations:** ^1^Division of the National Health Laboratory Service, National Institute for Communicable Diseases, Johannesburg, South Africa; ^2^Department of Clinical Microbiology and Infectious Diseases, School of Pathology, University of Witwatersrand, Johannesburg, South Africa; ^3^Infection Control Services Laboratory, Charlotte Maxeke Johannesburg Academic Hospital, National Health Laboratory Service, Johannesburg, South Africa; ^4^Microbiology Laboratory, Charlotte Maxeke Johannesburg Academic Hospital, National Health Laboratory Service, Johannesburg, South Africa; ^5^Pathcare/Vermaak Pathologists, Johannesburg, South Africa; ^6^Wits Donald Gordon Medical Centre, Johannesburg, South Africa; ^7^Department of Biochemistry, Genetics and Microbiology, Forestry and Agricultural Biotechnology Institute, University of Pretoria, Pretoria, South Africa

**Keywords:** *Acinetobacter baumannii*, colistin resistance, *lps*B gene, ST1, ST2, healthcare-associated bacteraemia, WGS, South Africa

## Abstract

*Acinetobacter baumannii* is an opportunistic pathogen and causes various infections in patients. This study aimed to describe the clinical, epidemiological and molecular characteristics of *A. baumannii* isolated from BCs in patients at a tertiary-level hospital in South Africa. Ninety-six isolates from bloodstream infections were collected. Clinical characteristics of patients were recorded from patient files. Organism identification and AST was performed using automated systems. PCR screening for the *mcr-1* to *mcr*-5 genes was done. To infer genetic relatedness, a dendrogram was constructed using MALDI-TOF MS. All colistin-resistant isolates (*n* = 9) were selected for WGS. The patients were divided into three groups, infants (<1 year; *n* = 54), paediatrics (1–18 years; *n* = 6) and adults (≥19 years; *n* = 36) with a median age of 13 days, 1 and 41 years respectively. Of the 96 *A. baumannii* bacteraemia cases, 96.9% (93/96) were healthcare-associated. The crude mortality rate at 30 days was 52.2% (48/92). The majority of the isolates were multidrug-resistant (MDR). All isolates were PCR-negative for the *mcr*-1 to *mcr*-5 genes. The majority of the isolates belonged to cluster 1 (62/96) according to the MALDI-TOF MS dendrogram. Colistin resistance was confirmed in nine *A. baumannii* isolates (9.4%). The colistin-resistant isolates belonged to sequence type (ST) 1 (5/6) and ST2 (1/6). The majority of ST1 isolates showed low SNP diversity (≤4 SNPs). All the colistin-resistant isolates were resistant to carbapenems, exhibited an XDR phenotype and harboured the *bla*_*OXA–*23_ gene. The *bla*_*NDM*_ gene was only detected in ST1 colistin-resistant isolates (*n* = 5). The *lps*B gene was detected in all colistin-resistant isolates as well as various efflux pump genes belonging to the RND, the MFS and the SMR families. The lipooligosaccharide OCL1 was detected in all colistin-resistant ST1 and ST2 isolates and the capsular polysaccharide KL3 and KL17 were detected in ST2 and ST1 respectively. This study demonstrated a 9.4% prevalence of colistin-resistant ST1 and ST2 *A. baumannii* in BC isolates. The detection of the *lps*B gene indicates a potential threat and requires close prospective monitoring.

## Introduction

*Acinetobacter baumannii* is a Gram-negative, non-fermenting opportunistic organism that causes a wide range of healthcare-associated infections, such as bacteraemia, meningitis, pneumonia, urinary tract infections, and wound infections (Hua et al., 2021). This organism has demonstrated resistance to multiple classes of antibiotics and there has been an increase in pan drug-resistant (PDR) and extensively drug-resistant (XDR) isolates in healthcare settings (Park et al., 2009; Gordon and Wareham, 2010). In many settings, colistin and tigecycline are the only remaining antibiotics available to treat multidrug-resistant (MDR) *A. baumannii* infections (Cai et al., 2012; Snyman et al., 2020). In South Africa, colistin is an unregistered drug and is used as salvage therapy for life-threatening infections (Majavie et al., 2021). Colistin use is widespread due to highly prevalent MDR and XDR *A. baumannii* infections in South African healthcare settings (Perovic and Chetty, 2016; Perovic et al., 2018; Ismail et al., 2019). An increase in colistin resistance is being reported in South Africa (Snyman et al., 2020; Majavie et al., 2021).

Colistin acts by modifying the negative charge of the outer membrane of *A. baumannii*, ultimately leading to the disruption of the bacterial membrane. The primary mechanisms of chromosomally-mediated colistin resistance include (i) the modification or loss of lipopolysaccharide (LPS) production due to mutations in any of the lipid A biosynthesis genes (i.e., *lpx*A, *lpx*C, and *lpx*D) and (ii) the modification of the PmrAB and PhoPQ two-component response regulator and sensor kinase system due to mutations in the *pho*PQ, *pmr*AB, and *mgr*B genes (Olaitan et al., 2014; Ahmed et al., 2016; Gogry et al., 2021). More recently, colistin resistance has been demonstrated through a plasmid carrying the mobile colistin resistance (*mcr*)-1 gene (Ahmed et al., 2016). A PubMed and Google Scholar literature search (date of search: 08 June 2022) for plasmid-mediated colistin resistance genes (i.e., *mcr*-1 to *mcr*-9) in clinical *Acinetobacter* spp. showed that the *mcr*-1 gene is already found in *A. lwoffii* strains in Italy and in *A. baumannii* strains in Iraq and Pakistan (Caselli et al., 2018; Hameed et al., 2019; Al-Kadmy et al., 2020; Hafudh et al., 2020). The *mcr*-2, *mcr*-3, and *mcr-4* genes are found in *A. baumannii* strains in Iraq and the *mcr*-4.3 gene is found in *A. baumannii* strains in Brazil, China and the Czech Republic as well as in *A. nosocomialis* strains in South Africa (Bitar et al., 2019; Ma et al., 2019; Al-Kadmy et al., 2020; Hafudh et al., 2020; Martins-Sorenson et al., 2020; Snyman et al., 2021). Colistin resistance has also been demonstrated through various efflux pumps (Ahmed et al., 2016; Gogry et al., 2021). Four classes of efflux pumps namely the major facilitator superfamily (MFS), the resistance-nodulation cell division (RND) family, the small multidrug resistance (SMR) family, and the multidrug and toxic compound extrusion (MATE) family are associated with antibiotic resistance in *A. baumannii* (Abdi et al., 2020). The RND class are the most important in MDR *A. baumannii* and colistin resistance has been associated with these efflux pumps (Ahmed et al., 2016; Abdi et al., 2020; Nogbou et al., 2022). The RND efflux pumps consist of a two-component regulatory system mediating the adaptive response of bacterial cells to a range of environmental stimuli (Ahmed et al., 2016).

Colistin resistance is a serious health threat worldwide and requires multisectoral research with proper surveillance and monitoring systems to report resistance, resistance mechanisms and associated genes (Gogry et al., 2021). Antimicrobial resistance surveillance reports from sentinel public hospitals in South Africa showed that colistin resistance in *A. baumannii* remained low over a period of 4 years [2012 = 3% (25/944); 2013 = 2% (28/1151); 2014 = 5% (29/624) and 2015 = 2% (20/819)] (Perovic et al., 2014a,b, 2015; Perovic and Chetty, 2016). Although colistin resistance is still low in South Africa it is still important to monitor and understand the mechanisms of colistin resistance given its current use as a last resort antibiotic. There are very few South African research papers published with a focus on colistin resistance and colistin resistance mechanisms; these studies could not determine the mechanisms of colistin resistance among circulating Gram-negative strains in South Africa (Newton-Foot et al., 2017; Snyman et al., 2020; Nogbou et al., 2022). This study aimed to describe and evaluate the clinical, epidemiological and molecular characteristics of *A. baumannii* isolated from blood cultures (BCs) in patients at a tertiary-level hospital in South Africa. Furthermore, all colistin-resistant isolates were characterised using whole-genome sequencing (WGS).

## Materials and methods

### Study setting and population

A prospective cross-sectional study was conducted in Gauteng, South Africa from October 2019 to December 2020 at a tertiary-level hospital (1,088 beds). The hospital offers specialist services, which include critical care units, paediatric and adult oncology, renal and liver transplant, and neonatology wards. The hospital is serviced by a 24-h, on-site Microbiology laboratory. Standard microbiological methods were used to identify bacterial pathogens. The VITEK^®^ 2 (bioMérieux, Marcy-I’Etoile, France) automated identification system was used to initially identify organisms at the Microbiology laboratory. Only *A. baumannii* isolated from BCs were considered for inclusion in this study.

A case of *A. baumannii* bacteraemia was defined as the first isolation of *A. baumannii* from BC. Healthcare-associated bacteraemia was defined as the collection of a positive BC > 48 h after hospital admission or when a patient had any healthcare contact 1 year before the current admission, including admission or referral from another healthcare facility. Community-associated bacteraemia was defined as the collection of a positive BC within 2 days of hospital admission and had no prior healthcare contact 1 year before the current admission.

Consent was obtained from adult patients for participation. In the case of a minor (≤18 years of age) informed consent was obtained from a parent or legal guardian. All clinical characteristics of patients were recorded from patient files. Laboratory information was obtained from the laboratory information system (LIS), TrakCare. Patient outcomes were recorded at the time of case report form (CRF) completion and followed-up 30 days after CRF completion (Supplementary Table 1).

### Phenotypic characterisation

All *A. baumannii* isolates that met the case definition were transported from the on-site Microbiology laboratory to the National Institute for Communicable Diseases (NICD), a division of the National Health Laboratory Service (NHLS), Gauteng, South Africa. Organism identification was confirmed with matrix-assisted laser desorption/ionization-time of flight mass spectrometry (MALDI-TOF MS) (Microflex, Bruker Daltonics, Leipzig, Germany). Antimicrobial susceptibility testing (AST) was performed using the Microscan Walkaway System with the NM44 card (Beckman Coulter, CA, United States). Colistin AST was confirmed with the Sensititre instrument (Trek Diagnostic Systems, East Grinstead, United Kingdom) using the FRCOL panel (Separation Scientific, Roodepoort, South Africa). The AST results were interpreted using the 2020 Clinical Laboratory Standards Institute (CLSI) guidelines (CLSI, 2020). MDR was defined as *A. baumannii* non-susceptibility to one or more antibiotic agents in three or more antimicrobial classes and XDR was defined as non-susceptibility to at least one agent in all but two or fewer antimicrobial classes (Magiorakos et al., 2012).

### Molecular characterisation

The total genomic DNA (gDNA) was extracted using a crude boiling method. Half a loop-full (∼1 μL) of subculture was re-suspended in tris-ethylenediaminetetraacetic acid (Tris-EDTA) buffer (10 mM:1 mM; pH 8) and heated at 95°C for 25 min. The lysed bacterial cells were discarded and the supernatant was harvested and stored at -20°C for further testing. A previously described multiplex PCR was used to identify the *mcr*-1 to *mcr*-5 genes (Rebelo et al., 2018). The amplicons were run on a 1.5% SeaKem LE agarose gel (Lonza, Basel, Switzerland) containing 5 μL of ethidium bromide (10 mg/mL) (Sigma-Aldrich, Burlington, VT, United States) at 100 V for approximately 2 h and visualised under a UV-light (G-box EF, Syngene, New Delhi, India).

A dendrogram was constructed with the MALDI-TOF MS instrument to determine the genetic relatedness of the isolates. This technique identifies bacterial isolates based on their unique protein profiles (Quainoo et al., 2017). A total of 96 *A. baumannii* isolates were sub-cultured onto MacConkey agar [Diagnostic Media Products (DMP), NHLS, Johannesburg, South Africa] and incubated overnight at 37°C. One colony of each of the cultured isolates was spotted onto a stainless-steel target plate (Bruker Daltonics, Leipzig, Germany) and was air-dried at room temperature. A volume of 1 μL of the matrix solution (consisting of alpha-cyano-4-hydroxycinnamic acid, absolute acetonitrile and trifluoroacetic acid) was placed onto each of the spotted samples. The solution and sample were allowed to co-crystallize and air-dried at room temperature. The prepared samples were analysed by using a MicroFlex LT mass spectrometer (Bruker Daltonics, Leipzig, Germany), which was operated using the MBT Compass (version 4.1) and flexControl software (version 3.4). Calibration was conducted using the bacterial test standard (Bruker Daltonics, Leipzig, Germany) as recommended by the manufacturer. The mass protein peaks of each spectrum were compared and a dendrogram was constructed. All distance values are relative and normalised to a maximal value of 1.6.

### Whole-genome sequencing and analysis

The gDNA of all colistin-resistant (MIC ≥ 4 mg/L) (CLSI, 2020) isolates (9/96) was extracted with the QIAamp mini kit (Qiagen, Hilden, Germany) with the inclusion of lysozyme (10 mg/mL; Sigma-Aldrich, Burlington, VT, United States) to ensure sufficient lysis. The quantity and quality of the extracted gDNA were determined on Qubit 4.0 (Thermo Scientific, Waltham, MA, United States). Multiplexed paired-end libraries were prepared using the Nextera DNA Prep kit, followed by sequencing (2 × 150 bp) on a NextSeq 550 instrument (Illumina Inc., San Diego, CA, United States) with 100× coverage at the NICD Sequencing Core Facility, NHLS, South Africa. Raw paired-end reads were analysed using the Jekesa pipeline (v1.0^1^). Briefly, Trim Galore! (v0.6.2^2^) was used to filter the paired-end reads (Q > 30 and length > 50 bp). *De novo* assembly was performed using SKESA (v2.3.0^3^) and the assembled contigs were polished using Shovill (v1.1.0^4^). Assembly metrics were calculated using QUAST (v5.0.2^5^). The assembled genome files were submitted to the National Center for Biotechnology Information GenBank and are available under BioProject number: PRJNA765178.

The multilocus sequence typing (MLST) profiles were determined using the MLST tool (version 2.16.4^6^). The core whole-genome sequence type (cgMLST) were determined using the “multiple genome analysis tools” from the BacWGSTdb (version 2.0^7^) (Feng et al., 2021). Virulence factors and antimicrobial resistance (AMR) gene search was performed using ABRicate (version 1.0.1^8^), against the Comprehensive Antibiotic Resistance Database (CARD), CARD-prevalence, Virulence Factor Database (VFDB) and ResFinder–Center for Genomic Epidemiology (CGE) database; with the gene alignment coverage cut-off of ≥95% and blastn sequence similarity of ≥95%. The haploid variants and single nucleotide polymorphisms (SNPs) were identified using Snippy (version 4.6.0^9^) against the *Acinetobacter baumannii* strain ab736 (NCBI accession: NZ_CP015121.1) reference genome; the default program parameters were used. The CGE web tool^10^ was used to construct a SNP comparison tree. The SNP comparison were exported as a Newick (NWK) file. The NWK file was uploaded to Microreact^11^ to visualise and edit the NWK tree (Argimon et al., 2016).

The genomic organisation of the antibiotic resistance genes and their genetic surrounds were compared against the closely related reference genome sequence using the whole genome comparison approach with Mauve (version 2.4.0^12^); the local collinear blocks (LCB) were identified using the progressive Mauve alignment method with the default parameters.

Using the draft genome sequences of the isolates, the surface polysaccharide loci and proteins were searched using Kaptive (version 2.0.0^13^) against the *A. baumannii* capsular polysaccharide locus (KL) and lipooligosaccharide outer core locus (OCL) databases (Wyres et al., 2020). Comparative diagrams were constructed using Clinker (version 0.0.23) (Gilchrist and Chooi, 2021).

### Statistical analysis

Microsoft Excel (version 2016) was used for data entry and basic statistical analysis. Additional data analyses were done using STATA statistical software package (version 14; StataCorp LP, TX, United States). Continuous data were summarised using the median and interquartile range (IQR). Univariate logistic regression was performed to explore factors associated with in-hospital mortality in infants. Variables were selected *a priori* based on their likelihood to contribute to mortality. *p*-values < 0.05 (two-tailed) were considered to be significant.

### Ethical approval

This study was approved by the Human Research Ethics Committee of the University of the Witwatersrand, Johannesburg, South Africa (protocol number: M180707).

## Results

### Clinical characteristics of patients

A total of 127 *A. baumannii* isolates were collected during the study period. Thirty-one isolates were excluded from the study (isolates could not be revived in the laboratory or belonged to other *Acinetobacter* spp., or no patient consent was obtained). The remaining 96 isolates came from patients who gave consent. The patients were divided into three groups, infants (<1 year; *n* = 54), paediatrics (1–18 years; *n* = 6) and adults (≥19 years; *n* = 36); the median age and interquartile range (IQR) were 13 days (IQR: 6–26 days), 1 year (IQR: 1–2 years) and 41 years (IQR: 30–50 years) respectively.

Of the 96 *A. baumannii* bacteraemia cases, 3.1% (3/96) were community-associated and 96.9% (93/96) were healthcare-associated (Table 1). The mean duration of admission when acquiring a healthcare-associated bloodstream infection was 16.9 days. Ninety-five point eight percent of the patients (92/96) received antimicrobial treatment during hospital admission. The most common antimicrobials prescribed was meropenem (83/96; 86.4%), colistin (41/96; 42.7%), gentamicin (32/96; 33.3%), and imipenem (21/96; 21.8%). The mean duration of antimicrobial treatment was 11.3 days. Thirty-seven point five percent (36/96) of the patients were admitted to the intensive care unit (ICU). The majority of patients presented with comorbidities (73.9%; 71/96) at the time of hospital admission. The HIV status was known for 89.6% (86/96) of all the patients. Only adult patients were HIV positive (16.3%; 14/86), for which the CD4 cell counts ranged from 0–755 cells/μL. Patient outcomes at the time of CRF completion were known for all patients (100%; 96/96) and 47.9% (46/96) of the patients died in the hospital. Patient outcome was known for 95.8% (92/96) of cases after a 30-day follow-up, and 52.2% (48/92) of patients died in the hospital. A higher proportion of male patients died [56.3% (27/48)]; possibly since there were more male patients admitted than female patients in this study. Peripheral intravenous lines and urinary catheters were the most common indwelling devices amongst all patient groups.

**TABLE 1:** Clinical characteristics of patients with *Acinetobacter baumannii* bacteraemia (October 2019 to December 2020).

Clinical characteristics	Infants: ≤1 year % (*n* = 54)	Paediatrics: 1–18 years % (*n* = 6)	Adults: ≥19 years % (*n* = 36)
**Sex**			
Male	53.7% (29)	66.7% (4)	61.1% (22)
Female	46.3% (25)	33.3% (2)	38.9% (14)
**Ethnicity**			
Black patients	94.4% (51)	100.0% (6)	97.2% (35)
Asian patients	-	-	2.8% (1)
Caucasian patients	5.6% (3)	-	-
**Infection origin**			
Community-associated	1.9% (1)	-	5.6% (2)
Healthcare-associated	98.1% (53)	100% (6)	94.4% (34)
**Prescribed antibiotics during admission**			
Yes	100.0% (54)	83.3% (5)	91.7% (33)
No	-	-	2.8% (1)
Unknown	-	16.7% (1)	5.6% (2)
**Admission to an intensive care unit**			
Yes	38.9% (21)	16.7% (1)	38.9% (14)
No	61.1% (33)	83.3% (5)	61.1% (22)
**Comorbidities**			
Yes*	58.3% (47)	50.0% (3)	58.3% (21)
No	11.1% (6)	33.3% (2)	25.0% (9)
Unknown	1.9% (1)	16.7% (1)	16.7% (6)
**HIV status**			
HIV negative	98.1% (53)	83.3% (5)	38.9% (14)
HIV positive	-	-	38.9% (14)
Unknown	1.9% (1)	16.7% (1)	22.2% (8)
**Crude in-hospital mortality after follow-up (30 days)**			
Alive#	55.6% (30)	66.7% (4)	38.9% (14)
Dead	44.4% (24)	33.3% (2)	61.1% (22)
**Clinical outcome after follow-up (30 days)**			
Deceased in the ICU	33.3% (18)	16.7% (1)	27.8% (10)
Deceased in the ward	11.1% (6)	16.7% (1)	33.3% (12)
Discharged from the hospital	33.3% (18)	50.0% (3)	16.7% (6)
Still admitted to the hospital	18.5% (10)	-	16.7% (6)
Transferred	1.9% (1)	-	-
Unknown	1.9% (1)	16.7% (1)	5.6% (2)
**Medical device used**†			
Yes^	53.7% (29)	83.33% (5)	97.2% (35)
No	46.3% (25)	16.67% (1)	-
**Type of medical device inserted (*n* = 80)**††	*n* = 29	*n* = 6	*n* = 45
Central venous catheter	-	-	15.6% (7)
Intra-arterial line	6.9% (2)	-	6.7% (3)
Peripheral intravenous line	89.7% (26)	8.3% (5)	31.1% (14)
Q-line	-	-	2.2% (1)
Urinary catheter	3.4% (1)	16.7% (1)	44.4% (20)

- = 0 or 0%; * = Patients that had any of the following conditions: anaemia, diabetes mellitus, epilepsy, hepatitis B, hypertension, malignancy, necrotizing fasciitis, prematurity, very low birth weight, renal transplant/dialysis or respiratory disease; # = Includes discharged patients; † = at the time of blood culture (BC) collection; ^ = 11 out of the 69 patients had two or more devices inserted at the same time; †† = The total (n = 80) includes all medical devices and not the number of case patients with devices.

### Mortality among *Acinetobacter baumannii* bacteraemia cases aged ≤1 year

The factors associated with in-hospital mortality at 30 days were reported for 53 out of 54 infant patients. Outcome data was unknown for one patient and were excluded from the univariate analysis. Age (*p* = 0.17), comorbidities (*p* = 0.15), gender (*p* = 0.53), treatment (*p* = 0.42) and type of medical device (*p* = 1.19) were not associated with in-hospital mortality.

### Phenotypic characterisation

**FIGURE 1:**
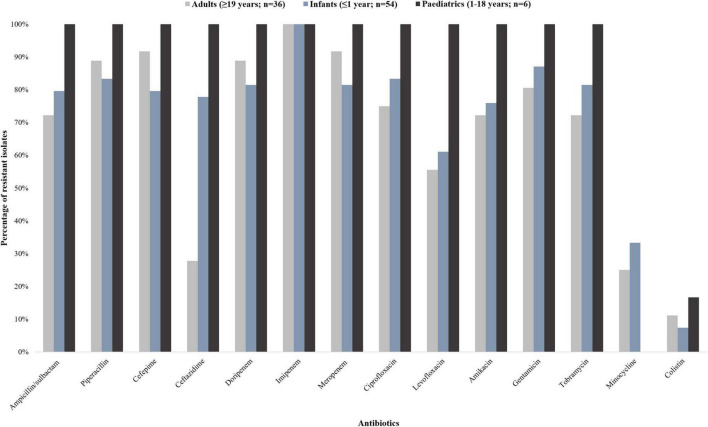
Antibiotic susceptibility profiles of 96 *Acinetobacter baumannii* isolates collected from patients with bacteraemia (October 2019 to December 2020). The 96 *A. baumannii* isolates were divided into three groups: infants (<1 year; *n* = 54), paediatrics (1–18 years; *n* = 6), and adults (≥19 years; *n* = 36). The interpretive categories of all the minimum inhibitory concentrations (MICs) were reported using the Clinical and Laboratory Standards Institute (CLSI) guideline.

A total of 96 *A. baumannii* isolates from BCs were included and processed in this study. The antibiotic susceptibility results are shown in Figure 1.

The isolates from infant patients showed more than 80% resistance toward ampicillin/sulbactam, cefepime, ciprofloxacin, doripenem, gentamicin, imipenem, meropenem, piperacillin, and tobramycin. The isolates from paediatric patients were resistant to all tested antibiotic classes except for minocycline and colistin (16.7% susceptibility; 83.3% intermediate susceptibility, respectively). The isolates from adult patients showed more than 80% resistance toward cefepime, doripenem, gentamicin, imipenem, meropenem, and piperacillin. Nine point four percent (9/96) of the isolates were resistant to colistin [having a minimum inhibitory concentration (MIC) breakpoint of ≥ 4 mg/L (CLSI)] (CLSI, 2020). Multidrug resistance was detected in 83.3% (45/54) and 91.7% (33/36) of the infant and adult patients respectively. Extensively drug resistance was detected in 100% (6/6) of the paediatric patients.

### Molecular characterisation

A total of 96 *A. baumannii* isolates were subjected to multiplex PCR screening of the *mcr*-1 to *mcr*-5 genes. All the isolates were PCR-negative.

**FIGURE 2:**
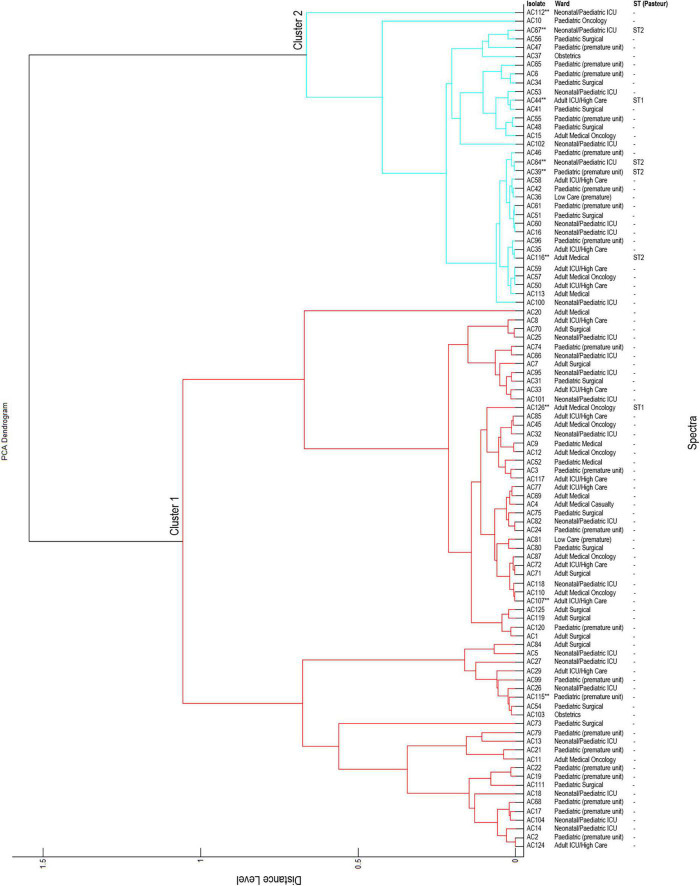
Principal component analysis (PCA) dendrogram generated by MALDI-TOF MS. **Colistin-resistant isolates; ST, sequence type; -, not determined. All distance values are relative and normalised to a maximal value of 1.6.

The constructed dendrogram showed that the *A. baumannii* isolates were grouped into two major clusters (Figure 2). Cluster 1 (red) and cluster 2 (blue) contained 62 and 34 isolates respectively. The majority of the *A. baumannii* isolates are grouped in cluster 1, except for a singleton AC20 (isolated from an adult patient). The remaining isolates are grouped in cluster 2, except for two singletons AC112 and AC10. Cluster 1 consisted of isolates from 35 infant patients, 26 adult patients and one paediatric patient. Cluster 2 consisted of isolates isolated from 19 infant patients, 10 adult patients and five paediatric patients. The majority of the colistin-resistant isolates (6/9; AC39, AC44, AC64, AC67, AC112, and AC116) are grouped in cluster 2 and the remaining colistin-resistant isolates (3/9; AC107, AC115, and AC126) are grouped in cluster 1.

Cluster 2 contained five colistin-resistant isolates (AC39, AC44, AC64, AC67, and AC116) belonging to sequence type (ST) 1 and cluster 1 contained one colistin-resistant isolate (AC126) belonging to ST2 (confirmed with WGS). WGS data was not available for isolates AC107, AC112 and AC115. It is speculated that isolate AC112 belongs to ST1 and isolates AC107 and AC115 belongs to ST2.

### Whole-genome sequencing

**FIGURE 3:**
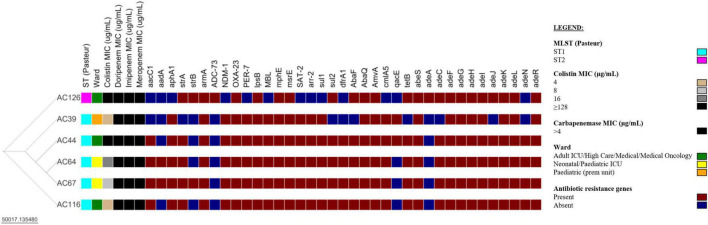
Comparison of six colistin-resistant *Acinetobacter baumannii* isolates. The Newick tree was visualised with Microreact version 103.0.0 (https://microreact.org/project/35sftMzdQgEKjds3t38re4-acinetobacter-baumannii-in-south-africa-2019-2020). The resistance genes were determined with the Comprehensive Antibiotic Resistance Database (CARD) version 3.2.2. The scale bar indicates the branch length; ST, sequence type; MIC, minimum inhibitory concentration; ICU, intensive care unit; prem unit, premature unit. See Supplementary Tables 3, 4 for additional information.

Whole-genome sequencing was performed on all nine colistin-resistant *A. baumannii* isolates. However, three (3/9) colistin-resistant *A. baumannii* isolates failed post-sequencing quality control (QC) and were excluded from further analysis. The six analysed isolates have a genome size ranging from 3.9–4.0 Mb and a GC content of 38.9–39.1% (Supplementary Table 2). The N50 values of the draft genomes of the colistin-resistant *A. baumannii* isolates were between 116,259–158,658 bp, with a sequencing depth of 89–123x. The comparison of the six colistin-resistant *A. baumannii* isolates is shown in Figure 3 and Supplementary Table 3. The colistin-resistant isolates belonged to ST1 (5/6) and ST2 (1/6) and were disseminated in multiple wards. The BacWGST database showed that isolates AC39, AC44, AC64, AC67, and AC116 belong to ST231 (Oxford MLST scheme)/ST1 (Pasteur MLST scheme) and the best reference genome is AYE_CU459141_ST231 which was isolated in France in 2001 (Genbank accession: CU459141); AC126 belongs to ST195 (Oxford MLST scheme)/ST2 (Pasteur MLST scheme) and the best reference genome is KAB01_CP017642_ST451 which was isolated in South Korea in 2016 (Genbank accession: CP017642). Colistin-resistant *A. baumannii* isolates belonging to ST1 (i.e., AC39, AC44, AC64, and AC67) were genetically indistinguishable with ≤ 4 SNPs (Supplementary Table 4). However, AC116 also belonging to ST1 differed from AC39, AC44, and AC67 with 101 SNPs and from AC64 with 103 SNPs. AC116 (ST1) and AC126 (ST2) showed high SNP diversity when compared to AC39, AC44, AC64, and AC67.

The six colistin-resistant *A. baumannii* isolates harboured multiple antibiotic resistance genes belonging to various antibiotic classes (Table 2). All six colistin-resistant *A. baumannii* isolates were resistant to carbapenems, exhibited an XDR phenotype and harboured the *bla*_*OXA–*23_ resistance gene. The IS*Aba*1 was detected upstream of the *bla*_*OXA–*23_ gene in isolates AC44, AC64, AC67, and AC116. The *bla*_*NDM*_ resistance gene was detected in all ST1 isolates (AC39, AC44, AC64, AC67, and AC116) (Supplementary Figure 1). The IS*Aba*125 was detected upstream of the *bla*_*NDM*_ in isolates AC39, AC44, AC67, and AC116. The colistin resistance-associated gene *lps*B was also detected in all six colistin-resistant isolates. None of the analysed colistin-resistant isolates harboured the *mcr* genes. Efflux genes belonging to the RND, the MFS and the SMR families were detected in the majority of our isolate collection (Table 2). The WGS results also showed that the *arm*A, *mph*E, and *msr*E antibiotic resistance genes were harboured together on the same contig in all six colistin-resistant isolates belonging to ST1 and ST2 (Supplementary Table 5). However, *aar*-2 and *cml*A1 resistance genes were harboured together on the same contig only in the ST1 isolates.

**TABLE 2:** Acquired and intrinsic antibiotic resistance genes and virulence genes associated with six colistin-resistant *Acinetobacter baumannii* isolates using whole-genome sequencing.

	Genes	CARD	
		ST1 (*n* = 5)	ST2 (*n* = 1)
**Antibiotic resistance**			
**Aminoglycoside**	*aac*C1	4	-
	*aad*A	2	-
	*aph*A1	5	-
	*str*A	4	1
	*str*B	1	1
	*arm*A	5	1
**Beta-lactam**	*NDM*-1	5	-
	*OXA*-23	5	1
	*PER*-7	5	-
**Polymyxin**	*lps*B	5	1
**Glycopeptide**	*BRP*	5	-
**Macrolide**	*mph*E	5	1
	*msr*E	5	1
**Nucleoside**	*SAT*-2	5	-
**Rifampicin**	*arr*-2	5	-
**Sulfonamide**	*sul*1	5	-
	*sul*2	4	1
**Trimethoprim**	*dfr*A1	4	-
**Efflux pumps**			
**Major facilitator superfamily**			
Fosfomycin	*Aba*F	4	1
Fluoroquinolone	*Aba*Q	5	1
Macrolide/Disinfecting agents and antiseptics	*Amv*A	5	1
Chloramphenicol	*cml*A5	5	-
Disinfecting agents and antiseptics	*qac*E	2	-
Tetracycline	*tet*B	4	1
**Small multidrug resistance**			
Aminocoumarin/Macrolide	*abe*S	5	1
**Resistance-nodulation-division AdeABC and AdeFGH**			
Glycylcycline/Tetracycline	*ade*A	-	1
Glycylcycline/Tetracycline	*ade*C	4	1
Fluoroquinolone/Tetracycline	*ade*F	5	1
Fluoroquinolone/Tetracycline	*ade*G	5	1
Fluoroquinolone/Tetracycline	*ade*H	5	1
Fluoroquinolone/Tetracycline	*ade*L	5	1
Glycylcycline/Tetracycline	*ade*R	5	1
**Resistance-nodulation-division AdeIJK**			
Beta-lactam/Fluoroquinolone/Lincosamide/Macrolide/Tetracycline	*ade*I	5	1
Beta-lactam/Chloramphenicol/Tigecycline/Quinolones	*ade*J	4	1
Beta-lactam/Fluoroquinolone/Lincosamide/Macrolide/Tetracycline	*ade*K	5	1
	*ade*N	4	-

The Comprehensive Antibiotic Resistance Database (CARD) version 3.2.2 was used; - = 0.

All six colistin-resistant isolates belonging to ST1 and ST2 share high similarity to the OCL1 region of the reference genome *A. baumannii* strain A1 (NCBI accession: CP010781.1) with 100.0% coverage and identity of ≥98.1% (Supplementary Figure 2). The KL regions identified in this study are clone specific. The KL17 was only identified in isolates belonging to ST1 (AC39, AC 44, AC64, AC67, and AC116) and shares similarities to the reference genome *A. baumannii* strain ATCC 17978 (NCBI accession: CP018664.1) with 100.0% coverage and identity of 99.9%. The KL3 was only identified in one isolate belonging to ST2 (AC126) and shares high similarity to *A. baumannii* strain Ab689 (NCBI accession: KC118541.2) with 100.0% coverage and identity of 98.8%.

## Discussion

*Acinetobacter baumannii* is ubiquitous in healthcare settings and causes a range of infections in patients. Infrequently, *A. baumannii* can also cause infections among individuals in the community. This study has shown that the majority of *A. baumannii* bloodstream infections are healthcare-associated. *Acinetobacter baumannii* was the most prevalent in infant patients (<1 year). Anane et al. (2020) also reported that *A. baumannii* infections were the most prevalent in paediatric patients (0–9 years) in the Eastern Cape, South Africa. However, another South African study conducted in a tertiary-level hospital in Gauteng from 2016 to 2017 showed that *A. baumannii* was most frequently isolated in adult patients (85.2%), followed by paediatric patients (62.5%) (Majavie et al., 2021). The overall crude in-hospital mortality rate in all patients was 47.9% at the time of CRF completion and increased to 52.2% after a 30-day follow-up. Majavie et al. (2021) reported an overall crude in-hospital mortality rate of 41.9% (18/43) in adult and paediatric patients. The mortality among paediatric patients in this study was high (45.2%; 24/53) compared to another South African study that reported a 20.9% mortality (9/43) among patients of the same age group (Majavie et al., 2021). The high mortality in this study could be because the majority of the patients were admitted to ICU or high care units, were infected with MDR *A. baumannii*, were severely sick and had co-morbidities.

Multidrug-resistant was detected in more than 80% of all the *A. baumannii* isolates. The high MDR rates are in accordance with other South African studies (Lowings et al., 2015; Lowe et al., 2018; Law et al., 2019; Von Knorring et al., 2019; Anane et al., 2020; Nogbou et al., 2022). The detection of nine colistin resistance isolates (9.4%) is worrisome as this is one of the last-resort antibiotics.

Based on the dendrogram constructed with the MALDI-TOF instrument, the *A. baumannii* isolates in this study are grouped into two major clusters. This suggests that colistin-resistant isolates share genetically similar attributes. Other genetic typing schemes such as MLST, pulsed-field gel electrophoresis (PFGE) and WGS are recommended as these schemes have higher discriminatory power compared to MALDI-TOF MS.

The six colistin-resistant *A. baumannii* isolates that were sequenced belonged to ST1 (5/6) and ST2 (1/6). In a global context, the BacWGST database showed that the six colistin-resistant are closely related to international strains from France and South Korea. This shows that ST1 and ST2 are global clones and have already been in circulation for several years.

All six colistin-resistant isolates were resistant to carbapenems and exhibited an XDR phenotype. Recently, colistin-resistant *A. baumannii* ST1 and ST2 isolates were reported in Brazil and South Africa; the authors also showed that the collected isolates were resistant to carbapenems and exhibited XDR phenotypes (Snyman et al., 2020; Carrasco et al., 2021; Nogbou et al., 2022). The most frequent β-lactamase genes detected in this study were *bla*_*OXA–*23_, *bla*_*NDM–*1_, and *bla*_*PER–*7_. Other South African studies also report a high frequency of *bla*_*OXA–*23_ in *A. baumannii* isolates which contributes to higher imipenem and meropenem resistance levels (Kock et al., 2015; Lowings et al., 2015; Lowe et al., 2018; Anane et al., 2020; Nogbou et al., 2022). The *bla*_*OXA–*23_ gene was associated with IS*Aba*1 (*n* = 4), this contributes to the overexpression and mobilisation of the *bla*_*OXA–*23_ gene as well as increased carbapenem resistance in *A. baumannii* (Nigro and Hall, 2016). The *bla*_*NDM*_ gene is detected to a lesser extent in South Africa (Anane et al., 2020). Our results which are supported by other published studies showed that the *bla*_*NDM*_ gene is clonal and only detected in ST1 *A. baumannii* isolates in South Africa (Snyman et al., 2020; Nogbou et al., 2022). The *bla*_*NDM*_ gene was associated with IS*Aba*125 (*n* = 4), which is associated with the horizontal transfer of *bla*_*NDM*_ in *A. baumannii* (Poirel et al., 2012). The colistin resistance-associated gene, *lps*B, was also detected in all our colistin-resistant isolates. Another South African study also reported the detection of the *lps*B gene in a colistin-resistant *A. baumannii* ST2 isolate (Nogbou et al., 2022). The six colistin-resistant isolates that underwent WGS were also negative for the *mcr*-1 to *mcr*-10 genes (all the tested isolates in this study were PCR negative for the *mcr*-1 to *mcr*-5 genes). Currently, in South Africa, colistin resistance is not attributed to any of the plasmid-borne *mcr* genes (Perovic and Chetty, 2016; Mahabeer et al., 2018; Snyman et al., 2020; Nogbou et al., 2022). It is nevertheless still important to monitor and test for the *mcr* genes in *A. baumannii* isolates as they can easily be transferred between bacterial species (Gogry et al., 2021). The upregulation and overexpression of antibiotic efflux pumps as shown in several studies could also play a role in colistin resistance (Peleg et al., 2008; Ahmed et al., 2016; Ni et al., 2016; Da Silva and Domingues, 2017; Machado et al., 2018; Karakonstantis, 2021; Nogbou et al., 2022). Efflux pumps, such as AdeABC and AdeFGH release various antibiotics including colistin from the bacterial cell which results in increased antibiotic resistance and tolerance (Ni et al., 2016; Machado et al., 2018; Abdi et al., 2020; Karakonstantis, 2021). Active screening and monitoring of efflux pumps are required as well as determining their role in colistin resistance. The precise mechanism/s of colistin resistance could not be determined in this study. It appears that colistin resistance is multifactorial. In addition, no other known colistin resistance genes or mutations were detected in our colistin-resistant *A. baumannii* isolates.

The surface polysaccharide loci and proteins were detected in the colistin-resistant isolates. The OCL1 was detected in all colistin-resistant *A. baumannii* isolates belonging to ST1 and ST2. Wyres et al. (2020) also reported the OCL1 in both ST1 and ST2 isolates but noted that OCL1 was the most predominant OCL type among ST2 *A. baumannii* genomes. KL3 (ST2) and KL17 (ST1) were clone specific and the findings in our study correlate with other previously reported results (Wyres et al., 2020). This indicates that genetic changes are taking place within the KL region and it differs between clones (Wyres et al., 2020).

Limitations of this study include: (i) small sample size; (ii) single-centre study therefore, data generated and reported here do not necessarily represent the overall epidemiology of *A. baumannii* in South Africa; (iii) three out of the nine colistin-resistant *A. baumannii* isolates did not pass sequencing QC and were excluded from further analysis (no funding was available for re-sequencing); (iv) no colistin-susceptible *A. baumannii* isolates from this study were sent for WGS, and (v) no functionality studies were performed to investigate resistance mechanisms in colistin-resistant *A. baumannii* isolates. Further studies are required to evaluate the factors involved in colistin resistance, mechanisms of acquisition and dissemination.

## Conclusion

The high prevalence of MDR *A. baumannii* isolates is a serious threat in South Africa, particularly in infants. This study demonstrated a 9.4% prevalence of colistin-resistant ST1 and ST2 *A. baumannii* BC isolates in the studied tertiary-level hospital. This is concerning given the lack of alternative antibiotic treatment options for MDR *A. baumannii* in this setting. The detection of the *lps*B gene indicates a potential threat and requires close prospective monitoring.

## Data availability statement

The dataset/s presented in this study can be found in the article or in the Supplementary material.

## Ethics statement

This study was approved by the Human Research Ethics Committee of the University of the Witwatersrand, Johannesburg, South Africa (protocol number: M180707). Consent was obtained from adult patients or their next of kin for participation. In the case of a minor (< = 18 years) informed consent was obtained from a parent or legal guardian.

## Author contributions

AS-M conceived and designed the study and wrote the first draft of the manuscript. ML performed the experiments and wrote the subsequent draft of the manuscript. ML, OP, TT, VC, TN, HI, and WL analysed the data. ML, WC, and AI analysed the colistin-resistant sequencing data. All authors were instrumental in the study coordination and the editing of the manuscript and read and approved the final manuscript.

## Footnotes

1
https://github.com/stanikae/jekesa

2
https://github.com/FelixKrueger/TrimGalore

3
https://github.com/ncbi/SKESA/releases

4
https://github.com/tseemann/shovill

5
http://quast.sourceforge.net/quast

6
https://github.com/tseemann/mlst

7
http://bacdb.cn/BacWGSTdb/

8
https://github.com/tseemann/ABRicate

9
https://github.com/tseemann/snippy

10
https://www.genomicepidemiology.org/services/

11
https://microreact.org/

12
https://darlinglab.org/mauve/mauve.html

13
https://github.com/katholt/Kaptive
