# Supplementary material for: Molecular characterisation of Acinetobacter baumannii isolates from bloodstream infections in a tertiary-level hospital in South Africa
Source: Front Microbiol. 2022 Aug 5;13:863129. doi: 10.3389/fmicb.2022.863129 (PMC9391000; doi:10.3389/fmicb.2022.863129)
Supplement: Supplementary file 1 [file Data_Sheet_1.PDF]

Supplementary Material – Molecular characterisation of *Acinetobacter baumannii* isolates from bloodstream infections in a tertiary-level hospital in South Africa

S1 Table Case Report Form (CRF)

Acinetobacter baumannii complex (Blood Culture Isolates only)

CRF COMPLETED BY: **SIGNATURE:** **DATE OF COMPLETION:** **CRF CHECKED BY:**

SOURCES OF DATA: Patient/Guardian □ Clinician □ Medical records □ No record found □ Not admitted □ Informed consent refused □

LABORATORY SPECIMEN NUMBER:

1. PATIENT DETAILS

1.1 Occupation: health care worker: Yes □ No □ Unknown □

1.2

Age: <1 month □ 1-3 months □ 3-6 months □ 6-9 months □

1.3 Gender: Male □ Female □

1.4. Race: Black □ Indian □ Asian □ Coloured □ White □

2. DETAILS OF CURRENT ADMISSION (RECORDS REVIEW)

2.2 Healthcare facility name: Province:

Was the patient referred/transferred from another hospital/chronic-care facility? Yes □ No □ Unknown □

If yes, specify which facility: ___________________

2.3 If hospitalised: Medical ward□ Paediatric ward □ Surgical ward□ Medical ICU□ Paediatric ICU□ Surgical ICU□ Obstetric ward□ Labour ward □

Outpatient: □ Neonatal ward □ Other □ If other, specify: ___________________

2.4 Date of admission: D D M M Y Y Y Y

2.5 Admission diagnosis:

2.6 Date of collection of specimen: D D M M Y Y Y Y

2.7 Date of *Aciba* complex result: D D M M Y Y Y Y

2.8 Antibiotics prescribed during this admission? Yes □ No □ Unknown □ *If yes, complete the table below*

Antibiotic name	Dosage (mg/g/IU)	(PO/IV IM)	Date started (enter date prescribed)	Date stopped	Duration of Treatment (days)
1			D D M M Y Y Y Y	D D M M Y Y Y	
2			D D M M Y Y Y Y	D D M M Y Y Y	
3			D D M M Y Y Y Y	D D M M Y Y Y	
4			D D M M Y Y Y Y	D D M M Y Y Y	
5			D D M M Y Y Y Y	D D M M Y Y Y	
6			D D M M Y Y Y Y	D D M M Y Y Y	
7			D D M M Y Y Y Y	D D M M Y Y Y	
8			D D M M Y Y Y Y	D D M M Y Y Y	

2.9. *A. baumannii* Directed Treatment

Antibiotic name	Dosage (mg/g/IU)	(PO/IV IM)	Date started (enter date prescribed)	Date stopped	Duration of Treatment (days)
1			D D M M Y Y Y Y	D D M M Y Y Y	
2			D D M M Y Y Y Y	D D M M Y Y Y	
3			D D M M Y Y Y Y	D D M M Y Y Y	
4			D D M M Y Y Y Y	D D M M Y Y Y	
5			D D M M Y Y Y Y	D D M M Y Y Y	
6			D D M M Y Y Y Y	D D M M Y Y Y	
7			D D M M Y Y Y Y	D D M M Y Y Y	
8			D D M M Y Y Y Y	D D M M Y Y Y	

2.10. Medical devices: Yes □ No □ Unknown □ If yes: Intravenous line □ Central venous catheter □ Urinary catheter □ Drainage port: □

Intra-arterial line □ Nasogastric tube □ Other □ Please specify: ____

Date of insertion for each device (including other) D D M M Y Y Y Y

Duration of device in situ on CRF completion: ________________________________

3. PAST MEDICAL AND RISK FACTORS

3.1 Underlying illness: Yes □ No □ Unknown □

If yes, give details: Diabetes □ Respiratory disease □ Renal transplant/dialysis □ CVS/Stroke □ Malignancy □

Very low/extremely low birth weight □ Prematurity □ Other □ Please specify: ____

3.2 HIV status: HIV positive□ HIV negative□ Unknown□ Patient/caregiver refuses to answer□

If positive, CD4 (cells/µl) count on admission: ______ Last CD4 count before this admission: ______
Source of HIV/CD4 results: Patient/caregiver □ DISA/Trakcare □

3.3. Previous admission. If "yes" is answered, please complete, the below table:

Adm No.	Healthcare facility name	Reason for admission	Type of medical care	Date of admission at the facility	Date of discharge from the facility	Days of admission
1.				D D M M Y Y Y	D D M M Y Y Y	
2.				D D M M Y Y Y	D D M M Y Y Y	
3.				D D M M Y Y Y	D D M M Y Y Y	
4.				D D M M Y Y Y	D D M M Y Y Y	

3.4 Antibiotic use in the last 6 months? Yes □ No □ Unknown □

If yes, was it during the previous hospitalisation: Yes □ No □
If known, list all the antibiotics in the last 6 months:

4. Is the organism a: Colonizer □ Pathogen □

5. SOURCE OF THE BACTERAEMIA (if pathogen)

5.1 CNS □ LRTI □ Gastrointestinal □ Bacteraemia with no focus □ Bone/Joint infection □ Skin/Soft tissue □ Abscess□ Surgery□ CVC □

Peripheral line □ if other -Specify: ___________________

6. SEVERITY OF ILLNESS ON THE DAY THE SPECIMEN WAS TAKEN

6.1Temp: ______ ˚c 6.2 BP: ______ / ______ 63 HR: ______

6.4 Resp rate: _____

6.5.Suppl oxygen: Yes □ No □ Unknown □

6.6 Mechanical ventilation: Yes □ No □ Unknown □

6.7 Mental status: Alert □ Sedated □ Disorientated □
Unconscious □ Stuporous □ Comatose □

6.8 Pitt Bacteraemia Score: _______________

6.9 Need for IV vasopressor support: Yes □ No □ Unknown □

6.10 Cardiac arrest: Yes □ No □ Unknown □

7. OUTCOME AT THE TIME OF COMPLETING CRF

7.1 Outcome: : Discharged from hospital (d/c) □ Discharged from ICU (d/c) □ Deceased in ward □ Deceased in ICU □ Still admitted □

RHT/Absconded □ Transferred □ Unknown □

If discharged or deceased Date of this outcome: D D M M Y Y Y Y

8. OUTCOME AT FOLLOW-UP (30 days after last positive *A. baumannii*)

8.1 Outcome: Discharged from the hospital (d/c) □ Discharged from ICU (d/c) □ Deceased in ward □ Deceased in ICU □ Still admitted □

RHT/Absconded □ Transferred □ Unknown □ Date of this outcome: D D M M Y Y Y Y

If in ICU, length of ICU stay: __________________

S2 Table Whole-genome sequencing statistics of six colistin-resistant *Acinetobacter baumannii* isolates

**Isolate number**	**AC39**	**AC44**	**AC64**	**AC67**	**AC116**	**AC126**
**Genome size (bp)**	3993106	3993776	3993533	3992240	4025100	3903999
**Number of contigs**	85	85	86	87	92	75
**Number of ORF**	3889	3890	3882	3882	3935	3788
**Coverage (x)**	103.035	89.202	123.213	94.564	93.071	95.456
**L75**	21	22	20	22	24	20
**N50 (bp)**	144865	134024	144765	144873	116259	158658
**GC (%)**	39.16	39.16	39.16	39.15	39.17	38.92

ORF = Open reading frames; bp = base pairs; L75 = The number of sequences evaluated at the point when the sum length exceeds 75% of the assembly size; N50 = 50% of the total sequence length

S3 Table Metadata of six colistin-resistant *Acinetobacter baumannii* isolates collected in 2020 in South Africa

(A) Clinical characteristics and antibiotic susceptibility results

ID	Collection date	ST (Pasteur)	Ward	Amikacin	Ampicillin/ Sulbactam	Cefepime	Ceftazidime	Ciprofloxacin	Doripenem	Gentamicin	Levofloxacin	Meropenem	Minocycline	Piperacillin	Tetracycline	Tobramycin	Trimethoprim/ sulfamethoxazole	Imipenem	Colistin
AC116	2020/10/10	1	Adult Medical	R	R	R	R	R	R	R	I	R	R	R	R	R	R	R	R
AC126	2020/11/27	2	Adult Medical Oncology	R	R	R	R	R	R	R	R	R	I	R	R	R	R	R	R
AC39	2020/01/10	1	Paediatric (premature unit)	R	R	R	R	R	R	R	R	R	I	R	R	R	R	R	R
AC44	2020/01/18	1	Adult ICU/ High Care	R	R	R	R	R	R	R	I	R	I	R	R	R	R	R	R
AC64	2020/03/07	1	Neonatal/ Paediatric ICU	R	R	R	R	R	R	R	R	R	I	R	R	R	R	R	R
AC67	2020/03/09	1	Neonatal/ Paediatric ICU	R	R	R	R	R	R	R	R	R	I	R	R	R	R	R	R

ST = Sequence Type; R = Resistant; I = Intermediate; S = Susceptible; The interpretive categories (R, I and S) were reported using the 2020 Clinical and Laboratory Standards Institute (CLSI) guideline

(B) Intrinsic and acquired resistance genes

ID	*aacC1*	*aadA*	*Aph A1*	*strA*	*StrB*	*armA*	*NDM-1*	*OXA-23*	*PER-7*	*lpsB*	*MBL/BRP*	*mphE*	*msrE*	*SAT-2*	*arr-2*	*sul1*	*sul2*	*dfrA1*
AC116	+	-	+	+	-	+	+	+	+	+	+	+	+	+	+	+	+	+
AC126	-	-	-	+	+	+	-	+	-	+	-	+	+	-	-	-	+	-
AC39	-	-	+	-	-	+	+	+	+	+	+	+	+	+	+	+	-	-
AC44	+	-	+	+	-	+	+	+	+	+	+	+	+	+	+	+	+	+
AC64	+	+	+	+	-	+	+	+	+	+	+	+	+	+	+	+	+	+
AC67	+	+	+	+	+	+	+	+	+	+	+	+	+	+	+	+	+	+

+ = Gene is present/detected; - = Gene is absent/not detected

(C) Efflux pumps

ID	*AbaF*	*AbaQ*	*AmvA*	*cmlA5*	*qacE*	*tetB*	*abeS*	*adeA*	*adeC*	*adeF*	*adeG*	*adeH*	*adeI*	*adeJ*	*adeK*	*adeL*	*adeN*	*adeR*
AC116	+	+	+	+	-	+	+	-	+	+	+	+	+	+	+	+	+	+
AC126	+	+	+	-	-	+	+	+	+	+	+	+	+	+	+	+	-	+
AC39	-	+	+	+	+	-	+	-	-	+	+	+	+	-	+	+	-	+
AC44	+	+	+	+	+	+	+	-	+	+	+	+	+	+	+	+	+	+
AC64	+	+	+	+	-	+	+	-	+	+	+	+	+	+	+	+	+	+
AC67	+	+	+	+	-	+	+	-	+	+	+	+	+	+	+	+	+	+

+ = Gene is present/detected; - = Gene is absent/not detected

Supplementary Table 4: **SNP differences between the six colistin-resistant *Acinetobacter baumannii* isolates (cut-off value ≤20 SNP differences)**

Isolate 1	Isolate 2	Matches	Mismatches	Jaccard.Index	Mash.like.distance	SNPs	SNP.distance
AC39	AC67	3980668	2986	0,99925	1,21E-05	1	2,51E-07
AC44	AC67	3980853	2928	0,999265	1,19E-05	1	2,51E-07
AC64	AC67	3981099	2588	0,99935	1,05E-05	1	2,51E-07
AC44	AC64	3981843	2028	0,999491	8,21E-06	2	5,02E-07
AC39	AC64	3981070	3262	0,999181	1,32E-05	3	7,54E-07
AC39	AC44	3981376	2498	0,999373	1,01E-05	4	1E-06
AC116	AC39	3928453	139990	0,965591	0,00057	101	2,57E-05
AC116	AC44	3928668	139872	0,965621	0,000569	101	2,57E-05
AC116	AC67	3927894	140492	0,965467	0,000572	101	2,57E-05
AC116	AC64	3928613	140134	0,965558	0,00057	103	2,62E-05
AC112	AC116	3522329	1735150	0,669965	0,007103	589	0,000167
AC112	AC39	3525008	1697834	0,674921	0,006961	638	0,000181
AC112	AC44	3525444	1697274	0,675021	0,006958	638	0,000181
AC112	AC64	3525385	1697544	0,674982	0,006959	639	0,000181
AC112	AC67	3525105	1697024	0,675032	0,006957	639	0,000181
AC112	AC126	3262674	2128843	0,60515	0,009108	5480	0,00168
AC112	AC115	2356225	10384141	0,184942	0,037557	15730	0,006676
AC115	AC44	2392721	9528549	0,20071	0,035344	18976	0,007931
AC115	AC64	2392884	9528375	0,200724	0,035342	18977	0,007931
AC115	AC39	2392822	9528035	0,200726	0,035342	18978	0,007931
AC115	AC67	2392765	9527533	0,20073	0,035341	18979	0,007932
AC115	AC116	2403049	9539539	0,201217	0,035276	19026	0,007917
AC115	AC126	2170089	9879842	0,180091	0,038282	19711	0,009083
AC107	AC112	2371332	7430954	0,241916	0,030409	20546	0,008664
AC116	AC126	2307770	3287697	0,412436	0,01735	21186	0,00918
AC126	AC39	2307126	3257027	0,414641	0,017228	21210	0,009193
AC126	AC64	2306741	3258261	0,414509	0,017235	21210	0,009195
AC126	AC44	2306684	3258223	0,414505	0,017236	21211	0,009195
AC126	AC67	2306502	3257659	0,414528	0,017234	21211	0,009196
AC107	AC115	2252324	13234799	0,145432	0,044216	22342	0,00992
AC107	AC126	2328408	6640231	0,259617	0,028587	22944	0,009854
AC107	AC116	2265342	6891980	0,24738	0,02983	23301	0,010286
AC107	AC39	2265236	6860234	0,248232	0,029741	23303	0,010287
AC107	AC67	2265116	6859858	0,248233	0,029741	23304	0,010288
AC107	AC44	2265070	6860878	0,248201	0,029744	23306	0,010289
AC107	AC64	2265090	6860990	0,2482	0,029744	23306	0,010289

Supplementary Table 5: Mobile genetic elements and their relationship to antibiotic resistance genes and virulence factors

Isolate	ST	Contig	Gene	Phenotype	Accession	Position in contig	Coverage (%)	Identity (%)
**AC39**	**1**	00001	*bla*OXA-69	amoxicillin, ampicillin	AY859527	226475-227299	100	100
		00030	*bla*ADC-25	unknown beta-lactam	EF016355	1223-74	99	96
		00044	*msr*E	erythromycin, azithromycin, virginiamycin s, pristinamycin ia, quinupristin	FR751518	2661-4136	100	100
		00044	*arm*A	amikacin, tobramycin, gentamicin, netilmicin, isepamicin	AY220558	6435-7208	100	100
		00044	*mph*E	erythromycin	DQ839391	1721-2605	100	100
		00048	*bla*NDM-1	cefepime, temocillin, cefoxitin, imipenem, cefixime, ertapenem, cefotaxime, amoxicillin+clavulanic acid, amoxicillin, piperacillin+tazobactam, meropenem, ceftazidime, piperacillin, ampicillin, ampicillin+clavulanic acid	FN396876	904-92	100	100
		00053	*dfr*A1	trimethoprim	X00926	1339-1812	100	100
		00053	*aad*A1	spectinomycin, streptomycin	JQ414041	1-662	83	100
		00061	*bla*PER-7	cefepime, cefoxitin, aztreonam, ticarcillin, cefotaxime, amoxicillin+clavulanic acid, ticarcillin+clavulanic acid, amoxicillin, piperacillin+tazobactam, ceftazidime, piperacillin, ampicillin, ampicillin+clavulanic acid	HQ713678	1018-92	100	100
		00062	*ARR*-2	rifampicin	HQ141279	1890-2342	100	100
		00062	*cml*A1	chloramphenicol	M64556	1569-310	100	99
		00063	*bla*OXA-23	imipenem, meropenem	AY795964	34-855	100	100
		00064	*aph*(6)-Id	streptomycin	M28829	1-712	85	100
		00066	*tet*(B)	doxycycline, tetracycline, minocycline	AP000342	1087-2292	100	100
		00069	*sul*2	sulfamethoxazole	AY034138	1376-2188	99	100
		00073	*aph*(3')-Ia	neomycin, lividomycin, ribostamycin, paromomycin, kanamycin	X62115	1570-755	100	100
		00074	*sul*1	sulfamethoxazole	U12338	4-843	100	100
		00074	*qac*E	cetylpyridinium chloride, ethidium bromide, chlorhexidine, benzylkonium chloride	X68232	1184-903	84	100
		00076	*aph*(3'')-Ib	streptomycin	AF321550	111-914	100	99
		00079	*aac*(3)-Ia	astromicin, fortimicin, gentamicin	X15852	4-537	100	98
**AC44**	**1**	00001	*bla*OXA-69	amoxicillin, ampicillin	AY859527	226475-227299	100	100
		00030	*bla*ADC-25	unknown beta-lactam	EF016355	1223-74	99	96
		00046	*msr*E	quinupristin, virginiamycin s, pristinamycin ia, erythromycin, azithromycin	FR751518	2661-4136	100	100
		00046	*arm*A	isepamicin, netilmicin, tobramycin, gentamicin, amikacin	AY220558	6435-7208	100	100
		00046	*mph*E	erythromycin	DQ839391	1721-2605	100	100
		00050	*bla*NDM-1	temocillin, ertapenem, cefepime, amoxicillin, ampicillin, imipenem, cefoxitin, piperacillin+tazobactam, cefixime, ampicillin+clavulanic acid, cefotaxime, amoxicillin+clavulanic acid, ceftazidime, meropenem, piperacillin	FN396876	904-92	100	100
		00052	*dfr*A1	trimethoprim	X00926	1339-1812	100	100
		00052	*aph*(6)-Id	streptomycin	M28829	4056-4780	86	100
		00052	*aad*A1	streptomycin, spectinomycin	JQ414041	1-662	83	100
		00063	*bla*PER-7	cefepime, amoxicillin, ticarcillin+clavulanic acid, ampicillin, cefoxitin, piperacillin+tazobactam, aztreonam, ticarcillin, ampicillin+clavulanic acid, cefotaxime, amoxicillin+clavulanic acid, ceftazidime, piperacillin	HQ713678	1018-92	100	100
		00064	*ARR*-2	rifampicin	HQ141279	1890-2342	100	100
		00064	*cml*A1	chloramphenicol	M64556	1569-310	100	99
		00065	*bla*OXA-23	imipenem, meropenem	AY795964	34-855	100	100
		00067	*tet*(B)	minocycline, doxycycline, tetracycline	AP000342	1087-2292	100	100
		00073	*aac*(3)-Ia	fortimicin, gentamicin, astromicin	X15852	1033-1566	100	98
		00074	*aph*(3')-Ia	lividomycin, kanamycin, paromomycin, ribostamycin, neomycin	X62115	1570-755	100	100
		00075	*sul*1	sulfamethoxazole	U12338	4-843	100	100
		00075	*qac*E	chlorhexidine, ethidium bromide, benzylkonium chloride, cetylpyridinium chloride	X68232	1184-903	84	100
		00076	*sul*2	sulfamethoxazole	AY034138	1139-327	99	100
		00077	*aph*(3'')-Ib	streptomycin	AF321550	111-914	100	99

**AC64**	**1**	00001	*bla*OXA-69	ampicillin, amoxicillin	AY859527	226475-227299	100	100
		00029	*bla*ADC-25	unknown beta-lactam	EF016355	1223-74	99	96
		00044	*msr*E	pristinamycin ia, quinupristin, virginiamycin s, erythromycin, azithromycin	FR751518	2661-4136	100	100
		00044	armA	netilmicin, isepamicin, gentamicin, amikacin, tobramycin	AY220558	6435-7208	100	100
		00044	mphE	erythromycin	DQ839391	1721-2605	100	100
		00048	*bla*NDM-1	ceftazidime, piperacillin, meropenem, cefoxitin, amoxicillin+clavulanic acid, cefotaxime, cefepime, imipenem, cefixime, ampicillin+clavulanic acid, ampicillin, piperacillin+tazobactam, temocillin, amoxicillin, ertapenem	FN396876	904-92	100	100
		00051	*bla*PER-7	ceftazidime, ticarcillin+clavulanic acid, aztreonam, piperacillin, ticarcillin, cefoxitin, amoxicillin+clavulanic acid, cefotaxime, cefepime, ampicillin+clavulanic acid, ampicillin, piperacillin+tazobactam, amoxicillin	HQ713678	1885-2811	100	100
		00061	*bla*OXA-23	meropenem, imipenem	AY795964	34-855	100	100
		00062	*ARR*-2	rifampicin	HQ141279	547-95	100	100
		00062	*cml*A1	chloramphenicol	M64556	2127-868	100	99
		00063	*aac*(3)-Ia	astromicin, fortimicin, gentamicin	X15852	1033-1566	100	98
		00065	*aph*(6)-Id	streptomycin	M28829	1-704	84	100
		00066	*tet*(B)	tetracycline, minocycline, doxycycline	AP000342	1087-2292	100	100
		00072	*aph*(3')-Ia	ribostamycin, lividomycin, neomycin, paromomycin, kanamycin	X62115	1570-755	100	100
		00074	*sul*2	sulfamethoxazole	AY034138	1139-327	99	100
		00075	*sul*1	sulfamethoxazole	U12338	13-852	100	100
		00077	*aph*(3")-Ib	streptomycin	AF321550	111-914	100	99
**AC67**	**1**	00001	*bla*OXA-69	ampicillin, amoxicillin	AY859527	226475-227299	100	100
		00030	*bla*ADC-25	unknown beta-lactam	EF016355	1223-74	99	96
		00046	*msr*E	azithromycin, erythromycin, pristinamycin ia, quinupristin, virginiamycin s	FR751518	2661-4136	100	100
		00046	*arm*A	isepamicin, amikacin, gentamicin, tobramycin, netilmicin	AY220558	6435-7208	100	100
		00046	*mph*E	erythromycin	DQ839391	1721-2605	100	100
		00050	*bla*NDM-1	cefixime, meropenem, piperacillin, ampicillin, ampicillin+clavulanic acid, ceftazidime, temocillin, imipenem, cefotaxime, amoxicillin+clavulanic acid, piperacillin+tazobactam, ertapenem, cefepime, cefoxitin, amoxicillin	FN396876	904-92	100	100
		00054	*bla*PER-7	piperacillin, ampicillin, ampicillin+clavulanic acid, ceftazidime, ticarcillin, cefotaxime, amoxicillin+clavulanic acid, piperacillin+tazobactam, cefepime, cefoxitin, ticarcillin+clavulanic acid, amoxicillin, aztreonam	HQ713678	1885-2811	100	100
		00062	*ARR*-2	rifampicin	HQ141279	1890-2342	100	100
		00062	*cml*A1	chloramphenicol	M64556	1569-310	100	99
		00064	*bla*OXA-23	meropenem, imipenem	AY795964	34-855	100	100
		00070	*aac*(3)-Ia	astromicin, fortimicin, gentamicin	X15852	1033-1566	100	98
		00071	*aph*(3")-Ib	streptomycin	AF321550	1725-922	100	99
		00071	*aph*(6)-Id	streptomycin	M28829	86-922	100	100
		00073	*aph*(3')-Ia	lividomycin, kanamycin, paromomycin, ribostamycin, neomycin	X62115	1244-429	100	100
		00075	*sul*2	sulfamethoxazole	AY034138	1139-327	99	100
		00077	*sul*1	sulfamethoxazole	U12338	4-843	100	100
**AC116**	**1**	00001	*bla*OXA-69	amoxicillin, ampicillin	AY859527	226475-227299	100	100
		00050	*msr*E	pristinamycin ia, quinupristin, virginiamycin s, erythromycin, azithromycin	FR751518	2661-4136	100	100
		00050	*arm*A	amikacin, netilmicin, gentamicin, tobramycin, isepamicin	AY220558	6435-7208	100	100
		00050	*mph*E	erythromycin	DQ839391	1721-2605	100	100
		00054	*bla*NDM-1	ertapenem, amoxicillin, temocillin, cefixime, meropenem, imipenem, cefepime, cefoxitin, ceftazidime, ampicillin, cefotaxime, amoxicillin+clavulanic acid, piperacillin+tazobactam, ampicillin+clavulanic acid, piperacillin	FN396876	904-92	100	100
		00060	*dfr*A1	trimethoprim	X00926	1339-1812	100	100
		00060	*aad*A1	streptomycin, spectinomycin	JQ414041	1-662	83	100
		00065	*bla*PER-7	amoxicillin, ticarcillin, cefepime, aztreonam, ticarcillin+clavulanic acid, cefoxitin, ceftazidime, ampicillin, cefotaxime, amoxicillin+clavulanic acid, piperacillin+tazobactam, ampicillin+clavulanic acid, piperacillin	HQ713678	1018-92	100	100
		00067	*bla*OXA-23	meropenem, imipenem	AY795964	34-855	100	100
		00068	*aph*(6)-Id	streptomycin	M28829	1-704	84	100
		00070	*ARR*-2	rifampicin	HQ141279	1660-2112	100	100

		00070	*cml*A1	chloramphenicol	M64556	1339-80	100	99
		00071	*tet*(B)	minocycline, doxycycline, tetracycline	AP000342	1087-2292	100	100
		00073	*sul*2	sulfamethoxazole	AY034138	1376-2188	99	100
		00077	*aac*(3)-Ia	astromicin, fortimicin, gentamicin	X15852	1033-1566	100	98
		00078	*aph*(3')-Ia	ribostamycin, lividomycin, paromomycin, kanamycin, neomycin	X62115	1360-545	100	100
		00079	*sul*1	sulfamethoxazole	U12338	4-843	100	100
		00080	*aph*(3")-Ib	streptomycin	AF321550	111-914	100	99
**AC126**	**2**	00005	*arm*A	gentamicin, amikacin, isepamicin, netilmicin, tobramycin	AY220558	191194-191967	100	100
		00005	*mph*E	erythromycin	DQ839391	195797-196681	100	100
		00005	*msr*E	erythromycin, azithromycin, pristinamycin ia, virginiamycin s, quinupristin	FR751518	194266-195741	100	100
		00010	*bla*OXA-66	unknown beta-lactam	AY750909	24632-25456	100	100
		00015	*bla*ADC-25	unknown beta-lactam	EF016355	1159-8	100	99
		00022	*aph*(3")-Ib	streptomycin	AF024602	59199-60001	99	100
		00022	*tet*(B)	minocycline, doxycycline, tetracycline	AP000342	64210-65407	99	100
		00022	*aph*(6)-Id	streptomycin	M28829	60001-60837	100	100
		00058	*bla*OXA-23	imipenem, meropenem	AY795964	34-855	100	100
		00064	*sul*2	sulfamethoxazole	AY034138	1440-625	100	100

Center for Genomic Epidemiology – Mobile Element Finder (software version: v1.0.3 and database version: v1.0.2); Antibiotic resistance genes harboured on the same contig are indicated in blue

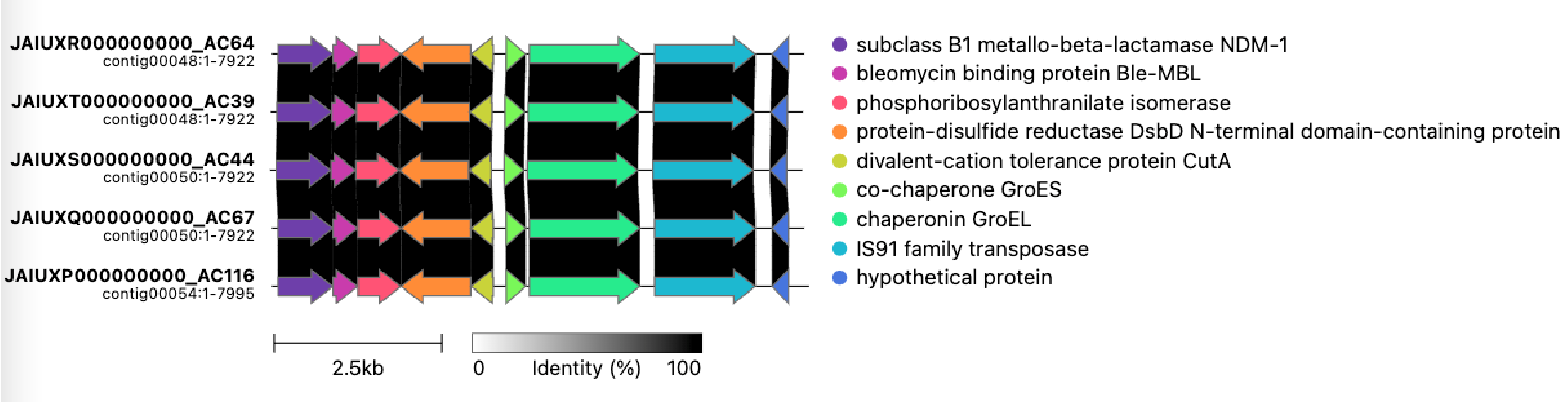

Supplementary Figure 1: Genome diagram representation of *Acinetobacter baumannii* surface polysaccharide loci

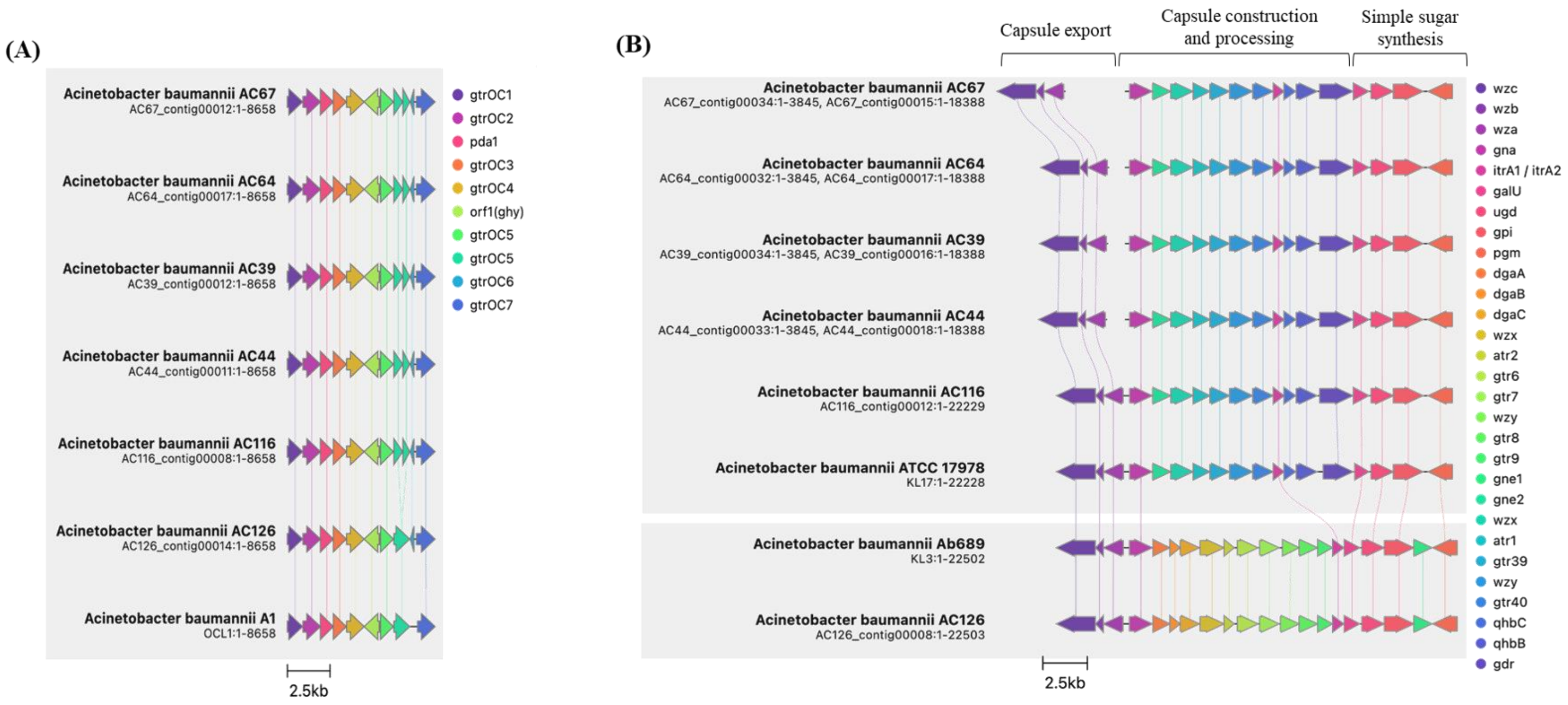

Supplementary Figure 2: Genome diagram representation of *Acinetobacter baumannii* surface polysaccharide loci

Genes are represented by arrows orientated in the direction of transcription that is coloured according to the schemes shown on the right-hand side of (A) and (B).

(A) Comparison of lipooligosaccharide outer core synthesis loci of the isolates loci to the lipooligosaccharide outer core locus (OCL)-1 from the Kaptive Database;

(B) Comparison of capsular polysaccharide loci (KL) between the isolates to the reference loci KL-3 and KL-17 from the Kaptive Database. The gaps/grey spaces between the genes as shown in (B) do not represent missing genetic material, such as genes.
